# Plasma Choline Concentration Was Not Increased After a 6-Month Egg Intervention in 6–9-Month-Old Malawian Children: Results from a Randomized Controlled Trial

**DOI:** 10.1093/cdn/nzab150

**Published:** 2022-02-23

**Authors:** Megan G Bragg, Elizabeth L Prado, Charles D Arnold, Sarah J Zyba, Kenneth M Maleta, Bess L Caswell, Brian J Bennett, Lora L Iannotti, Chessa K Lutter, Christine P Stewart

**Affiliations:** Institute for Global Nutrition, Department of Nutrition, University of California Davis, Davis, CA, USA; Institute for Global Nutrition, Department of Nutrition, University of California Davis, Davis, CA, USA; Institute for Global Nutrition, Department of Nutrition, University of California Davis, Davis, CA, USA; Institute for Global Nutrition, Department of Nutrition, University of California Davis, Davis, CA, USA; School of Public Health and Family Medicine, Kamuzu University of Health Sciences, Blantyre, Malawi; USDA Western Human Nutrition Research Center, Davis, CA, USA; USDA Western Human Nutrition Research Center, Davis, CA, USA; Brown School, Institute for Public Health, Washington University in St Louis, St Louis, MO, USA; RTI International, Washington DC, USA; Institute for Global Nutrition, Department of Nutrition, University of California Davis, Davis, CA, USA

**Keywords:** choline, trimethylamine *N*-oxide, LMIC, growth, development, complementary foods

## Abstract

**Background:**

Eggs are a rich source of choline, an essential nutrient important for child growth and development. In a randomized trial of 1 egg/d in young children in Ecuador, an egg intervention led to significant improvements in growth, which were partially mediated by increased plasma choline concentration. A similar trial in Malawi (clinicaltrials.gov: NCT03385252) found little improvement in child growth or development.

**Objectives:**

We aimed to evaluate the effect of 1 egg/d for 6 mo on plasma choline concentrations in Malawian children enrolled in a randomized trial.

**Methods:**

Infants aged 6–9 mo in rural Malawi were randomly assigned to receive 1 egg/d (*n *= 331) or serve as a nonintervention control (*n *= 329) for 6 mo. Anthropometric, developmental, and dietary data were collected at baseline and 6-mo follow-up, along with a blood draw. Plasma choline, betaine, dimethylglycine, trimethylamine *N*-oxide (TMAO), and DHA were measured at both time points using ultrahigh performance liquid chromatography–tandem MS (*n *= 200 per group). Linear regression analysis was used to determine the difference in plasma choline and related metabolites between groups after 6 mo of intervention.

**Results:**

Plasma choline, betaine, dimethylglycine, and DHA concentrations did not differ between groups at 6-mo follow-up. Plasma TMAO was significantly (26%; 95% CI: 7%, 48%) higher in the egg intervention group in a fully adjusted model.

**Conclusions:**

Provision of 1 egg/d for 6 mo did not result in increases in plasma choline or related metabolites, except TMAO. This could partially explain the lack of effect on growth and development. Additional interventions are needed to improve choline status, growth, and development in this population.

## Teaser

Provision of 1 egg/d for 6 mo did not result in increases in plasma choline or related metabolites, except TMAO, in this randomized trial of young Malawian children.

## Introduction

Choline is an essential nutrient needed for optimal child growth and development, especially memory development (1). Animal foods are the main sources of choline, but are relatively expensive (2). Therefore, choline intake is likely low in many low- or middle-income countries (LMICs) (3). Suboptimal intake of choline in the pre- and postnatal periods can put children at risk of poor growth and development.

Eggs are a rich source of choline, along with other nutrients important in early life (4). In Ecuadorian infants age 6–9 mo at baseline, significant improvements in growth were measured after provision of 1 egg/d for 6 mo compared with controls (5). Plasma concentrations of choline, betaine, trimethylamine *N*-oxide (TMAO), and DHA were also increased in the egg group, and the improvement in length-for-age *z*-score (LAZ) was partially mediated by increased plasma choline concentration (6). However, a similar study performed in Malawi found no significant effects on growth, except larger head-circumference-for-age *z*-score (HCAZ) in children in the egg group (7). Measures of child development were included in the Malawian study, with mostly null results, except for a smaller percentage of children with fine motor delays in the egg intervention group (8).

It is unclear why the egg intervention had so little effect on growth in Malawi compared with Ecuador. Reported adherence was high in the Malawian trial. Eggs were reportedly consumed by 71% of children in the egg group on a 24-h dietary recall performed at the 6-mo follow-up (7). However, dietary recall data revealed inadequate intakes of several nutrients, including choline, even with frequent egg consumption (9). It is unclear whether the increase in choline intake in the Malawian trial was sufficient to improve plasma choline concentration. Considering the mediating role of plasma choline in improvement of growth in Ecuador, the effect of the egg intervention on plasma choline in the Malawian study could help explain the drastic difference in results.

The objective for this analysis was to evaluate the effect of provision of 1 egg/d for 6 mo on plasma choline concentration in Malawian children aged 6–9 mo at baseline. We hypothesized that children in the egg intervention group would have higher plasma choline compared with children in the control group. This is a secondary outcome of the main Mazira Project randomized trial. We also tested for group differences in several related metabolites: betaine (a methyl donor and the oxidized product of choline), dimethylglycine (DMG, a metabolite of betaine), TMAO (produced from choline by gut microbiota), and DHA (another nutrient important for child growth and development and linked to choline metabolism) (10). In secondary exploratory analyses, we tested several sociodemographic factors as moderators in the relation between intervention group and plasma choline or its metabolites, as well as the role of plasma choline or its metabolites as mediators for the 2 primary outcomes that were significantly different by intervention group (HCAZ and fine motor delay). Finally, in order to provide evidence of adherence to the egg intervention, preliminary metabolomics analyses of the intervention effect are presented.

## Methods

### Study design

The Mazira Project (clinicaltrials.gov: NCT03385252) was a randomized trial of 660 Malawian children that took place from February 2018 to January 2019. The primary growth outcomes of the trial have been published previously (7). Infants aged 6–9 mo were individually randomly assigned to intervention or control for 6 mo. Randomization occurred using a 1:1 allocation ratio in blocks of 10, based on a random sequence generated by a researcher independent from the field team. Study staff read a description of the trial to all caregivers and then met individually with caregivers to review the consent statement and respond to questions. If the caregiver agreed to participate, staff invited caregivers to choose 1 sealed envelope, which contained the allocation code, under monitoring by an independent community member. Families in the intervention group received weekly egg deliveries, with instructions to feed 1 egg/d to the enrolled child. Eggs were procured from a local distributor. On average, eggs weighed 53 g and provided 126 mg choline (9). Families in the control group were asked to feed the child his or her typical diet. Descriptions of food and nutrient intakes for study participants have been published (9, 11); generally, complementary diets were maize-based, with some inclusion of green leafy vegetables but limited intake of animal-source foods. Both groups received instruction on food hygiene and handwashing during home visits.

At each clinic visit, all participants received incentive gifts that included household goods, such as fabric for clothing, wash basins, buckets, and cooking utensils, as well as cash reimbursement for travel costs. In addition, at study completion, participants in the control group were given a package of goods of equal value to the 6 mo of eggs provided to the intervention group. After the study, results were communicated to participants at community meetings, and participants were given the opportunity to share their experiences. In addition, study results were presented to the Mangochi District Health Committee, nutrition leaders at the Ministry of Health, and the egg producer who supplied eggs to the project. Study protocols were reviewed and approved by institutional review boards at the University of California Davis and the College of Medicine in Malawi.

### Participants

Singleton infants aged 6–9.9 mo residing in the Lungwena Health Center and St Martin's Rural Hospital in Malindi catchment areas in Mangochi District were eligible to participate. These areas are low-income with high rates of food insecurity, similar to other rural areas in Malawi. Study staff identified age-eligible infants using listings generated by community health workers and recruited them during household visits. Infants were excluded for egg allergy, history of serious allergic reactions requiring emergency care, congenital defects or conditions that affect growth and development, severe anemia (hemoglobin <5 g/dL),  low midupper arm circumference (MUAC; <12.5 cm), presence of bipedal edema, acute illness or injury warranting hospital referral, or if the family planned to leave the area in the next 6 mo. Infants with acute illness or injury, low hemoglobin or MUAC, or bipedal edema were referred to the local health center. Costs of care were paid by the study, and the infant was eligible for reassessment for entry to the study after the illness had resolved.

### Data collection

Study staff, who were blinded to group assignment, collected an array of dietary, anthropometric, and sociodemographic data during study visits at enrollment and at 6-mo follow-up. Anthropometric data were converted to *z*-scores using WHO growth standards (12), with the cutoff of *z*-score ≤ −2 used to define stunting (length-for-age), underweight (weight-for-age), wasting (weight-for-length), and low head circumference (head-circumference-for-age). The Malawi Developmental Assessment Tool (MDAT) was administered at enrollment and 6-mo follow-up. This tool measures fine motor, gross motor, personal social, and language development using a series of pass/fail tasks. Developmental delay in each domain was defined as failing ≥2 tasks that 90% of children at the same age would pass, using a Malawian reference population. The MDAT has been validated for use in Malawi and has high sensitivity (97%) and specificity (82%) to detect neurodevelopmental impairment in this context (13).

Soon after enrollment, staff visited the home to collect information about housing materials and animal ownership for the creation of a housing and asset index. During the same home visit, staff administered the Household Food Insecurity Access Scale (14). Additionally, caregivers reported morbidity symptoms at weekly home visits. Detailed information about data collection procedures is available in prior publications (7, 8).

### Plasma analysis

Study nurses collected venous blood into lithium heparin tubes at enrollment and 6-mo follow-up. Venous whole blood samples were used to identify anemia (Hemocue 201; HemoCue Inc; anemia defined as hemoglobin <11 g/dL) and malaria antigens (DF Bioline Malaria Ag P.f/Pan; Abbott Diagnostics). Afterwards, samples were centrifuged at room temperature at 3000 rpm for 15 min within a mean 28 ± 42 min of collection and aliquoted and stored in a local −20°C freezer within a mean 37 ± 14 min of centrifugation. Each afternoon, aliquots were transported for storage in a laboratory facility at −80°C.

Plasma choline was quantitated using 2 independent assays. First, it was included in a semiquantitative metabolomics analysis by Metabolon Inc. For this analysis, 200 children per group (*n *= 400) with adequate blood samples at enrollment and follow-up were randomly selected for inclusion. More than 800 other metabolites were measured, including plasma betaine, DMG, TMAO, and DHA. After precipitation of proteins with methanol, addition of recovery standards, and centrifugation, sample extracts were inserted onto a C18 column (Waters UPLC BEH C18-2.1 × 100 mm, 1.7 µm) and flushed with water, methanol, 0.05% perfluoropentanoic acid, and 0.1% formic acid. Samples were dried and then reconstituted in solvent containing standards at fixed concentrations. Ultrahigh performance liquid chromatography–tandem mass spectrometry (UPLC-MS/MS) was performed using a Waters ACQUITY ULPC with a Thermo Scientific Q-Exactive high-resolution mass spectrometer, paired with a heated electrospray ionization (HESI-II) source and Orbitrap mass analyzer. Injection order of the samples was randomized on the instrument and quality of the run was monitored using evenly spaced control samples and internal and recovery standards. Metabolites were identified by Metabolon Inc using authenticated standards. For data measured over several days, the median was set to 1.00 for each run-day block, and data points were normalized proportionally to adjust for day-to-day instrument variation. Missing values were imputed with the minimum. These data are semiquantitative and reported in “relative intensity” units, which provide information about the distribution of plasma choline as well as within-study comparisons. However, because these data are not reported in absolute concentrations, they cannot be compared with other studies.

To determine the absolute concentrations, a second, targeted quantitative assay of plasma choline, betaine, and TMAO was performed in a subsample (*n *= 60) of participants randomly chosen from the metabolomics sample. These analyses were performed at the USDA Western Human Nutrition Research Center using LC-MS/MS as described by Wang et al. (15) with modifications. DMG was not included in these analyses. Briefly, 20-µL plasma samples were aliquoted to a 2-mL Eppendorf tube and mixed with 80 µL 10 µM surrogate standard (deuterated analytes in methanol) then vortexed for 30 s and centrifuged at 18,000 × *g* at 10°C for 10 min. Supernatant was transported to glass inserts in HPLC vials. Similarly, standards were produced from 0 µM to 100 µM of nondeuterated analytes in methanol. Then, 20 µL of each standard and 80 µL of 10 µM surrogate standard were transferred to 150-µL glass inserts in HPLC vials. All standards were purchased from Sigma-Aldrich. All reagent solvents were purchased from Fisher Scientific and were MS grade. Samples (5 µL) were injected onto a silica column (2.0 × 150 mm, 5-µm Luna silica; Cat. No. 00F-4164-B0; Phenomenex) at a flow rate of 0.25 mL/min using a Waters Acquity UPLC outfitted with an API 4000 Q-TRAP mass spectrometer (AB SCIEX). Then, a discontinuous gradient of 0.1% acetic acid in water with 0.1% acetic acid in methanol at varying ratios was introduced. Electrospray ionization in positive-ion mode with multiple reaction monitoring was used to measure analytes. Integration and quantification of values was completed using Analyst 1.6.2 software. Linear regression models were used to calculate standard linearity. These data were used to estimate the mean concentration of plasma choline, betaine, and TMAO in our study. However, due to the small sample size, they were not used for intervention group comparisons.

Additionally, plasma C-reactive protein (CRP) and α-1 acid glycoprotein (AGP) were measured using ELISA by the VitMin lab in Germany (16).

### Statistical analysis

A detailed statistical analysis plan was developed prior to analysis and posted publicly (https://osf.io/azf7q/). The main outcome was the difference in mean plasma choline between groups after 6 mo of intervention, as calculated in linear regression models using intention-to-treat analysis. Minimally adjusted analyses included baseline plasma choline values as a covariate. Fully adjusted analyses were adjusted for sociodemographic factors, including: child age, sex, and birth order; maternal age, height, education, occupation, literacy, marital status, tribe, and religion; housing and asset index; animal ownership (chickens, cows, and goats); food insecurity score; distance to water source; number of children aged <5 y in the household; and village location (Lungwena compared with Malindi health center catchment areas). Additionally, month of blood sample collection and time since last intake of foods other than breast milk, water, or tea were considered. Covariates were retained in the model if they were associated with plasma choline with *P* < 0.1. Each model was assessed for normality and homoscedasticity of residuals using the Shapiro–Wilk and Breusch–Pagan tests, respectively. Nonnormal variables were log transformed. Outliers were assessed using boxplots, histograms, scatterplots and Cook D; outliers that were considered impossible were corrected or removed before analysis. Similar analyses were performed to test for group differences in plasma betaine, DMG, TMAO, and DHA.

A sample size of 200 per group is sufficient to detect an intervention effect on plasma choline and its metabolites as small as 0.28 SDs with 80% power and an α of 0.05. This effect size is similar to the effect on plasma choline demonstrated in the egg intervention trial in Ecuador (0.35; 95% CI: 0.12, 0.57) (6).

In exploratory analyses, the following characteristics were assessed as moderators in the relation between group assignment and plasma metabolites: child sex, birth order, baseline maternal age and education, baseline household food insecurity, baseline housing and asset index, and baseline LAZ. Each moderator was tested as a dichotomous variable by including an interaction term in the minimally adjusted models. Additionally, for the 2 main effects that were significant in primary analyses (HCAZ and fine motor delay), structural equation modeling was used to assess the mediating role of plasma choline and related metabolites.

To investigate whether there was metabolomic evidence of adherence to the egg intervention, the metabolites from the semiquantitative UPLC-MS/MS metabolomics analysis were analyzed using linear regression models to estimate mean difference between groups, adjusting for the baseline metabolite concentration and child age and sex, using intention-to-treat analysis. Metabolites with >20% of samples missing were excluded from the analysis; in general, these were metabolites of medications or other chemicals that were expected to be missing in this infant population. Normality was checked using a Shapiro–Wilk test statistic cutoff of 0.95. Nonnormal metabolites were log transformed, and ranked data were used if normality was still not achieved. Outcomes were summarized with geometric means and CIs regardless of transformation used for analysis. Fold changes were calculated as the ratio of geometric means. Volcano plots and *P*-value histograms were used to assess the overall intervention effect, and heatmaps were used to explore patterns of significance among metabolic pathways. Although there are a large number of outcomes, only the uncorrected *P* values were reported because these analyses were exploratory in nature. The statistical analysis plan for the semiquantitative metabolomics analysis was developed a priori and is published at https://osf.io/r6amq/.

All analyses were 2-sided tests with an α of 0.05. Given the number of exploratory tests, significant *P* values should be interpreted with caution.

## Results

### Participant characteristics

**FIGURE 1:**
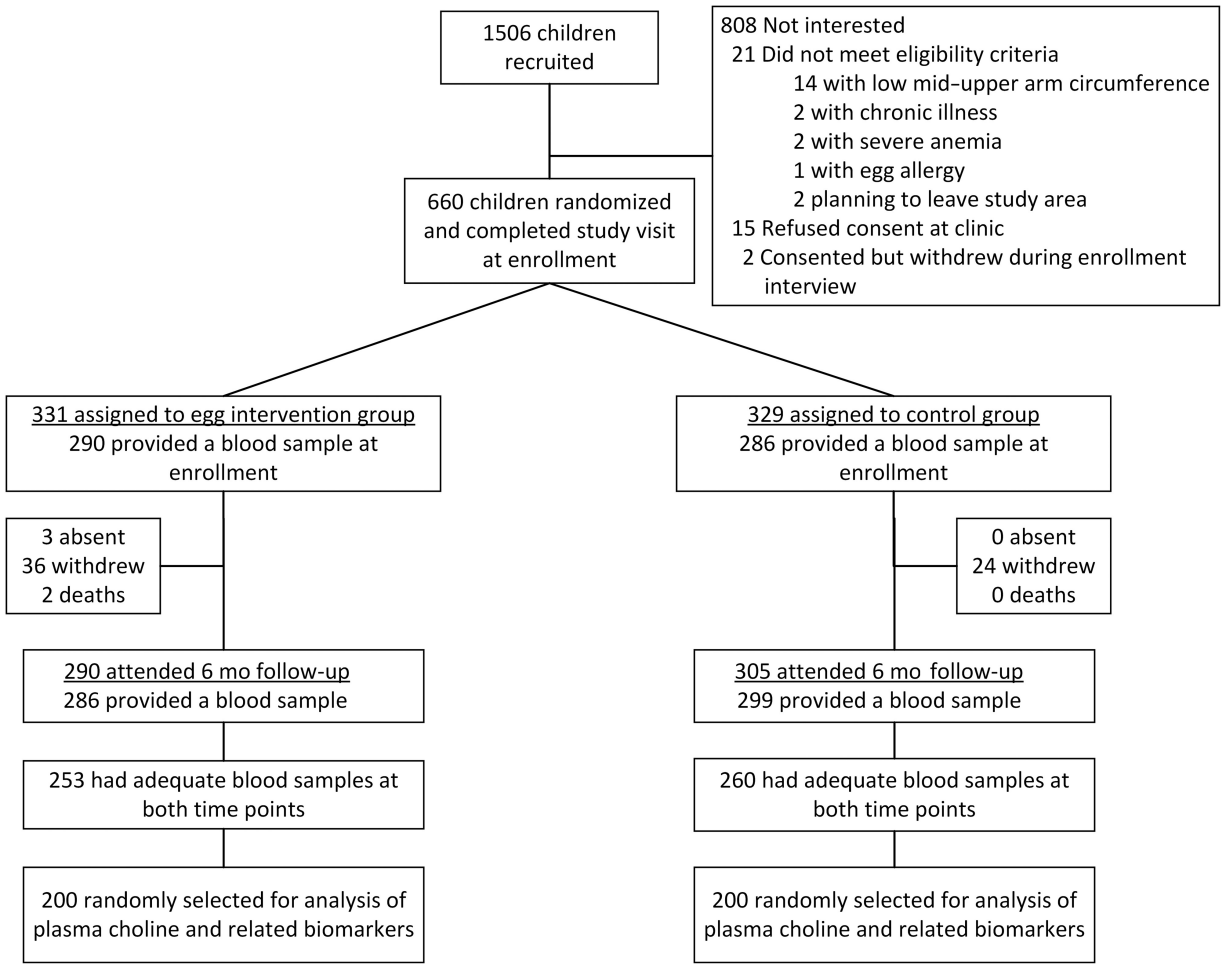
Participant flow diagram for this secondary analysis of the Mazira Project.

This analysis included 200 children per group (**Figure 1**). At baseline, infants in both groups were similar in age, consumption of animal-source foods, and measures of growth and development (**Table 1**). There were higher levels of maternal education in the egg group, with more mothers having completed primary school and able to read compared with the control group. In addition, households in the egg intervention group were more likely to own chickens; however, chicken ownership was not significantly associated with plasma choline values.

**TABLE 1:** Baseline characteristics of Mazira Project participants included in this analysis (*n *= 400)^1^

	Intervention (*n* = 200)	Control (*n* = 200)
Maternal and child characteristics		
Child age, mo	7.5 ± 1.2	7.3 ± 1.2
Male, %	53.5	53.0
First born, %	31.2	24.5
Animal-source food consumption,^2^ %		
Consumed dairy	10.6	9.0
Consumed meat	3.5	1.5
Consumed fish	27.1	20.0
Consumed eggs	3.5	4.0
Any breast milk, %	99.5	100.0
Prevalence of anemia (Hgb <11 g/dL), %	62.3	62.0
Prevalence of malaria, %	14.0	15.0
Prevalence of stunting (LAZ ≤ −2), %	13.5	14.0
Prevalence of wasting (WLZ ≤ −2), %	1.5	1.5
Prevalence of underweight (WAZ ≤ −2), %	6.5	10.0
Prevalence of small head size (HCAZ ≤ −2), %	21.5	25.5
Prevalence of developmental delay,^3^ %		
Gross motor	0.5	0.0
Fine motor	2.5	2.6
Personal social	1.5	1.5
Language	0.5	0.5
Maternal age, y	26.0 ± 6.6	26.0 ± 6.9
Maternal BMI, kg/m^2^	21.7 ± 3.3	21.8 ± 2.7
Mother completed primary school, %	28.5	22.5
Mother can read, %	52.8	42.7
Household characteristics		
Number of household members	5.8 ± 2.5	6.2 ± 2.8
Moderate to severe food insecurity,^4^ %	77.5	78.5
Owns latrine, %	95.5	97.5
Owns cow(s), %	2.5	2.5
Owns goat(s), %	17.0	23.0
Owns chicken(s), %	28.0	38.5
Less than 10 min to water source, %	55.3	51.8

1 Values are means ± SD or percentages. HCAZ, head-circumference-for-age *z*-score; Hgb, hemoglobin; LAZ, length-for-age *z*-score; WAZ, weight-for-age *z*-score; WLZ, weight-for-length *z*-score.

2 As reported by the caregiver on a 24-h dietary recall.

3 As defined by the Malawi Developmental Assessment Tool (13).

4 As defined by the Household Food Insecurity Access Scale (14).

Children included in this analysis were similar to those in the main trial who were excluded from analysis (*n* = 260) (**Supplemental Table 1**), with some exceptions. Among excluded infants, there was a lower prevalence of malaria (8.6% compared with 14.5%, *P* = 0.042) and small head size (HCAZ ≤ −2: 15.1% compared with 23.5%, *P* = 0.008), and a higher prevalence of fish consumption at baseline (32.7% compared with 23.6%, *P* = 0.010).

The semiquantitative (*n *= 400) and quantitative (*n *= 60) choline measures were well correlated (*r* = 0.92, **Supplemental Figure 1**), as were the betaine (*r* = 0.98) and TMAO (*r* = 0.98) measurements. Plasma choline and betaine concentrations were similar between groups at baseline (*n *= 60; **Table 2**), whereas plasma TMAO was higher in the control group at baseline. Plasma choline decreased across the study period, whereas plasma betaine and TMAO concentrations increased.

**TABLE 2:** Mean plasma concentrations of selected nutrients at enrollment and 6-mo follow-up in participants in the Mazira Project egg intervention trial^1^

	Baseline				Six-month follow-up			
	Intervention		Control		Intervention		Control	
	*n*	Mean ± SD	*n*	Mean ± SD	*n*	Mean ± SD	*n*	Mean ± SD
Plasma choline, µmol/L	30	17.0 ± 3.3	30	17.2 ± 3.8	30	15.5 ± 4.3	30	13.7 ± 2.6
Plasma betaine, µmol/L	30	86.3 ± 34.8	30	83.8 ± 28.4	30	101.6 ± 34.3	30	91.7 ± 35.1
Plasma TMAO,^2^ µmol/L	30	1.0 (0.6, 2.9)	30	2.3 (1.3, 4.7)	30	3.8 (2.5, 6.7)	30	3.3 (1.8, 4.5)

1 TMAO, trimethylamine *N*-oxide.

2 Variable was skewed; data presented are median (IQR).

### Intervention effect on plasma choline and related metabolites

At 6-mo follow-up, plasma choline and betaine were higher and plasma DHA was lower in the egg intervention group compared with control; however, the differences were not statistically significant (*n *= 400; **Table 3**). Plasma TMAO was higher in the egg intervention group, an effect that remained significant after adjustment. Because plasma TMAO was log transformed, the fully adjusted β value of 0.23 (95% CI: 0.07, 0.39) represents a 26% (95% CI: 7%, 48%) increase in TMAO in the egg intervention group compared with control. Adjustment for covariates did not substantively change the results. Plasma TMAO was not significantly associated with markers of inflammation (CRP: *r* = 0.42, *P* = 0.41; AGP: *r* = 0.17, *P* = 0.17).

**TABLE 3:** Minimally and fully adjusted group differences in plasma choline and selected nutrients at 6-mo follow-up in young Malawian children enrolled in the Mazira Project egg intervention trial (*n *= 400)^1^

	Minimally adjusted^2^		Fully adjusted^3^	
	Estimate (95% CI)^4^	*P* value	Estimate (95% CI)^4^	*P* value
Plasma choline	0.15 (−0.03, 0.34)	0.11	0.17 (−0.02, 0.35)	0.08
Plasma betaine	0.07 (−0.09, 0.24)	0.38	0.09 (−0.07, 0.26)	0.28
Plasma DMG^5^	−0.01 (−0.07, 0.05)	0.75	−0.01 (−0.07, 0.05)	0.66
Plasma TMAO^5^	0.26 **(**0.10, 0.43**)**	0.002	0.23 (0.07, 0.39)	0.01
Plasma DHA	−0.07 (−0.27, 0.12)	0.45	−0.05 (−0.24, 0.15)	0.62

1 DMG, dimethylglycine; TMAO, trimethylamine *N*-oxide.

2 Adjusted for baseline plasma values.

3 Additionally adjusted for child age, sex, and birth order; maternal age, height, education, occupation, literacy, marital status, tribe, and religion; housing and asset index; animal ownership; food insecurity score; distance to water source; number of children aged <5 y in the household; village location (Lungwena compared with Malindi health center catchment areas); month of data collection; and time since last intake of food or drink. Covariates were included in the model if they were associated with plasma variable with *P* < 0.1.

4 Estimates are β coefficients representing the effect of egg intervention group assignment, in SD units. These estimates use relative intensity data, and cannot be directly translated to effect per absolute concentration (i.e., micromoles per liter).

5 Outcome variable was log transformed. Estimates represent the difference in the means of the log outcome; after exponentiation, this value can be interpreted as the ratio of the geometric means.

### Effect modification and mediation exploratory analyses

Seven variables were tested as effect modifiers in the relation between intervention group and plasma metabolites. Of 35 tests for effect modification (**Supplemental Table 2**), none were significant at the 0.05 level.

Plasma metabolites were also tested as mediators in the relation between intervention group and the 2 outcomes that were significant in primary intervention effect analyses (fine motor delay and HCAZ). Tests for mediation were similarly null, with no significant mediation by plasma choline or related metabolites (**Supplemental Table 3**).

### Semiquantitative metabolomics analysis

A total of 689 metabolites were included in the analysis after excluding those metabolites for which >20% of samples were missing, and 87 metabolites had a statistically significant difference between the egg and control groups (*n *= 400, **Supplemental Figure 2**). Visualizing the data using a volcano plot (**Supplemental Figure 3**), 22 metabolites were identified as both statistically significant (*P *< 0.05) and having a large-magnitude fold change (log_2_ fold change >0.25). Eleven of the 22 metabolites, including TMAO, were part of the lipid metabolic pathway, and 4 were part of the amino acid pathway (**Table 4**).

**TABLE 4:** Metabolites for which there was a statistically significant intervention effect with large-magnitude fold change after Malawian children consumed 1 egg/d for 6 mo (*n *= 400)^1^

Code	Biochemical^2^	Superpathway	Subpathway	*P* value	Fold change^3^
x40473_6	Hydantoin-5-propionate	Amino acid	Histidine metabolism	0.016	0.821
x57687_6	*N*,*N*,*N*-Trimethyl-5-aminovalerate	Amino acid	Lysine metabolism	0.003	1.21
x541_6	4-Hydroxyphenylacetate	Amino acid	Phenylalanine metabolism	0.028	1.23
x27672_6	3-Indoxyl sulfate	Amino acid	Tryptophan metabolism	0.015	1.30
x37092_6	γ-Glutamyl-2-aminobutyrate	Peptide	γ-Glutamyl amino acid	0.001	1.27
x48425_6	Phenylacetylcarnitine	Peptide	Acetylated peptides	0.006	1.35
x35126_6	Phenylacetylglutamine	Peptide	Acetylated peptides	0.003	1.30
x396_6	Glutarate (C5-DC)	Lipid	Fatty acid dicarboxylate	0.046	1.21
x54907_6	Hexanoylglutamine	Lipid	Fatty acid metabolism (acyl glutamine)	0.003	0.834
x40406_6	Trimethylamine *N*-oxide	Lipid	Phospholipid metabolism	0.004	1.25
x35625_6	1-Myristoyl-glycerol	Lipid	Monoacylglycerol	0.002	0.787
x32506_6	2-Linoleoyl-glycerol	Lipid	Monoacylglycerol	0.009	0.827
x54966_6	Diacylglycerol*	Lipid	Diacylglycerol	0.014	0.816
x57373_6	Palmitoyl-docosahexaenoyl-glycerol*	Lipid	Diacylglycerol	0.010	0.838
x37198_6	5α-Pregnan-3β,20α-diol disulfate	Lipid	Progestin steroids	0.000	1.33
x32425_6	Dehydroepiandrosterone sulfate (DHEA-S)	Lipid	Androgenic steroids	0.008	1.27
x38168_6	16α-Hydroxy DHEA 3-sulfate	Lipid	Androgenic steroids	0.019	1.19
x63731_6	Glycoursodeoxycholic acid 3-sulfate	Lipid	Secondary bile acid metabolism	0.009	1.38
x601_6	Dihydroorotate	Nucleotide	Pyrimidine metabolism, orotate containing	0.006	0.818
x15753_6	Hippurate	Xenobiotics	Benzoate metabolism	0.014	1.37
x62533_6	(2,4 or 2,5)-Dimethylphenol sulfate	Xenobiotics	Food component/plant	0.017	0.793
x48698_6	6-Hydroxyindole sulfate	Xenobiotics	Chemical	0.025	1.32

1 Metabolites with statistically significant *P* values (*P* < 0.05) from linear regression models adjusted for baseline measures of the outcome variables and child age and sex, and large-magnitude fold changes (log_2_ fold change >0.25), which were calculated as the ratio of geometric means. Biochemical compound codes, names, superpathways, and subpathways provided by Metabolon Inc.

2 An asterisk (*) indicates compounds that have not been officially confirmed based on a standard, but for which Metabolon Inc is confident in the identity.

3 Fold changes for which the intervention egg group had a higher mean are >1, and fold changes for which the control group had a higher mean are <1.

## Discussion

In this sample of Malawian children, plasma choline was not significantly increased after a 6-mo intervention of 1 egg/d compared with a control. Mean plasma choline concentrations were ∼17 µmol/L at 6–9 mo of age, and ∼14 µmol/L at 12–15 mo of age, and did not differ by group. Similarly, neither plasma betaine, DMG, nor DHA were increased with the intervention, although plasma TMAO was significantly higher in the egg intervention group. In exploratory analyses, none of the plasma metabolites significantly mediated the primary intervention effects on growth or development.

Our findings contrast with the previous egg intervention trial in Ecuador (the Lulun Project), in which plasma choline, betaine, TMAO, and DHA were significantly higher in the group receiving 1 egg/d, with effect sizes ranging from 0.29 SD for betaine to 0.43 SD for DHA. The concentrations of plasma choline, betaine, and TMAO were similar (within ∼15% of each other) across studies, after pooling Lulun Project values across groups and converting to micromoles per liter (6). The eggs provided in both studies were rich sources of choline (6, 9). Given the identical intervention and the increased sample size, which provided more power to detect intervention effects, we expected to detect similar improvements in choline status in our trial.

However, there were differences between the trials that might explain these findings. First, background choline intake might have varied between study sites. Choline intake was not reported in the Lulun Project, but the mean intake in infants in our trial was only 102 mg/d at baseline (9), far below the Adequate Intake (AI) level for their age (AI for 7–12 mo: 150 mg/d; 1–3 y: 200 mg/d) (17). Intake remained low, despite the egg intervention: at the 6-mo follow-up, 97% of children in the intervention group and 100% of children in the control group had choline intakes below the AI (9). Intake in Ecuador might have been higher, because the prevalence of egg consumption was higher at baseline (∼40% compared with ∼4% in Malawi) (5, 7), providing a rich source of choline even before the intervention began. Consumption of breast milk was common at both sites, although neither breast milk intake nor choline concentrations were measured. Breast milk choline concentrations can vary with genotype and maternal choline supplementation (18), although there was no difference in breast milk water-soluble choline concentrations between Canadian and Cambodian women (19). Overall, it is possible that background choline intake was higher in Ecuador, even if this was not reflected by higher plasma choline values. Plasma choline concentration might not reflect small to moderate changes in intake (20). Plasma choline is homeostatically regulated, with increased recycling through phosphatidylcholine and decreased oxidation to betaine during deficiency (21). Perhaps in the context of very low dietary choline intake in Malawi, the addition of 1 egg/d was below the threshold needed to significantly change plasma choline concentrations. However, choline requirements might have differed by study site. Choline metabolism is affected by common single nucleotide polymorphisms (SNPs) (22), and there is evidence for negative selection of SNPs related to choline requirements in African populations with traditionally low choline intake (23). Genotype was not measured in either study; perhaps in Malawi, a reduced prevalence of these SNPs led to lower choline needs than in the Lulun Project and therefore reduced the potential to benefit from a choline-rich intervention.

Background consumption of DHA might also have differed between trials. Fish consumption was more prevalent in our trial, with ∼25% of children consuming fish at baseline and ∼65% at 6-mo follow-up [compared with ∼17% and ∼20% in the Lulun Project (5, 7)]. Of note, fish was not a major source of choline intake in our trial due to the small portions consumed and the lower choline content compared with eggs and breast milk (24). Although infant portion sizes of fish were small (∼20 g) (11), breast milk of women near Lake Malawi is rich in DHA due to maternal fish consumption (25). DHA is thought to act synergistically with choline to improve neural development (10). Given their synergy, high DHA seems unlikely to have prevented increases in plasma choline; however, the high background intake might explain why plasma DHA was not significantly increased by the egg intervention in Malawi.

The significant difference in plasma TMAO between intervention and control groups at 6-mo follow-up is intriguing. Besides conversion from choline, TMAO can be directly consumed (i.e., in fish) or converted from carnitine. Plasma TMAO increased in both groups across the study period, perhaps due to increased consumption of fish and other complementary foods as children aged. However, there was no difference in intake of flesh foods (including fish) between groups at follow-up (11), suggesting direct intake of TMAO was not different between groups. One explanation is that conversion of choline to TMAO in the gut caused increased production of TMAO in the group receiving eggs. This might be related to a third factor that likely differed between study sites: the composition of the gut microbiome, which influences conversion of choline to TMAO (26, 27). Although not measured in either study, differences in diet, environmental pathogens, and access to sanitation between Malawi and Ecuador could lead to differences in the gut microbiome. Perhaps in Malawi, there was more preferential conversion of choline to TMAO than in Ecuador, which prevented increases in plasma choline. It is also possible that this finding is due to chance, given the number of statistical tests. Although TMAO is linked to atherosclerosis and inflammation in adults (28), its role in the health of young children is unknown. In our study sample, there was no link between plasma TMAO and markers of inflammation, and plasma TMAO concentrations were lower than those linked to poor health outcomes in adults (29). Similarly, a study of healthy 11-y-old children in Australia found no association between TMAO and inflammatory biomarkers or indicators of metabolic syndrome (30). Given the unclear biological significance of plasma TMAO in young children, this finding does not warrant restriction of egg intake during the complementary feeding period.

To our knowledge, no other studies besides the Lulun and Mazira Projects have evaluated change in plasma choline after an egg intervention in young children; however, several studies of egg consumption have been conducted in adults. The majority of these studies reported increases in plasma choline without changes in plasma TMAO with daily consumption of eggs (31–34). However, 1 small study noted a significant increase in plasma TMAO within hours of ingesting eggs, with large interindividual differences in change in TMAO (35). There is a need for more studies of the effects of eggs on concentrations of plasma choline and related metabolites, especially in young children in diverse settings. The current study area was similar to other rural Malawian communities, although the prevalence of wasting and stunting was lower compared with the latest national data (1.5% and ∼14%, respectively, compared with 3% and 37%) (36). Our study could be generalizable to young children in LMICs with a similar background diet (low consumption of animal-source foods, except fish) and socioeconomic status.

Given the lack of effects on growth, development, and plasma choline concentration in the Mazira Project, the question of adherence becomes important. We monitored adherence in 3 ways. First, we collected replicate 24-h dietary recalls at baseline, 3 mo, and 6 mo. Second, we administered weekly FFQs throughout the trial. For both of these methods, egg intake was reported by caregivers, rather than directly observed; thus, the potential for reporting bias is a limitation to adherence estimates. There are currently no metabolites that have been identified as biomarkers of egg intake (37). Given the lack of an objective standard, 24-h dietary recalls are recommended as the best option for examining the effect of an intervention on intake (38). Although exploratory, the 22 metabolites identified in the semiquantitative metabolomics analysis as being impacted by group assignment provide a third measure of adherence to the egg intervention. These metabolites also provide insight into potential markers of egg consumption and metabolic pathways that might be sensitive to changes in dietary egg intake in children. One recent study in adult men using nontargeted metabolic profiling found several metabolites associated with egg intake (39). Although there were no shared metabolites identified between our study and that one, the major metabolic pathways that were impacted in our study (amino acid, peptide, and lipid) are not unexpected given the nutrient content of eggs. In both trials, few metabolites with direct links to egg compounds (such as those involved with choline metabolism) were impacted, suggesting a broad influence of egg intake on metabolism. Our findings could be used in future egg intervention studies to identify metabolites to target for analyses.

Our analysis has several strengths, including a randomized design with low losses to follow-up, as well as measurement of several metabolites related to choline in a relatively large sample of 400 children. Also, analyses were prespecified in a statistical analysis plan. However, it also has several weaknesses. First, growth and development, not plasma choline concentration, were the primary outcomes for this trial. The trial was not specifically designed to isolate the effect of a specific nutrient, although plasma choline was specified as an a priori secondary outcome (40). Also, adherence was self-reported and might be influenced by reporting bias. Even with self-report, compliance with the egg intervention was not complete. However, the metabolomics analysis provides suggestive evidence that true adherence was high enough to cause measurable changes. Finally, plasma choline might not be a reliable biomarker of choline status, because it is insensitive to small to moderate changes in intake (20), and represents only a small fraction of choline found in the body (21). Many studies, including ours, include measurements of choline metabolites, such as betaine and DMG, as a way to clarify choline status. Choline can also be converted to acetylcholine, phosphatidylcholine, and sphingomyelin; however, these metabolites were not included in this study. Identification of sensitive biomarkers of choline status is an active area of research (41). Future studies with a broader suite of biomarkers could help reveal the relation between egg intake and choline status.

In conclusion, unlike in a similar trial in Ecuador, our trial of 1 egg/d did not result in increases in plasma choline or its metabolites, except plasma TMAO, in young Malawian children. These contrasting findings can help explain differences in the primary growth outcomes, because choline was responsive to intake and mediated effects on growth in Ecuador. Additional interventions are needed to improve choline status and growth in this population. More research is needed to understand the role of eggs on choline status in young children in diverse contexts, including identification of sensitive biomarkers of choline status.

## Supplementary Material

nzab150_Supplemental_File

## Data Availability

The data underlying this article are available on the Mazira Project OSF page: https://osf.io/vfrg7/.

## Notes

The Mazira Project randomized trial was funded by the Bill & Melinda Gates Foundation (OPP1169033). The funder had no role in study design, data analysis, or preparation of the manuscript.

Author disclosures: The authors report no conflicts of interest. MGB is funded by the Egg Nutrition Center.

Supplemental Figures 1–3 and Supplemental Tables 1–3 are available from the “Supplementary data” link in the online posting of the article and from the same link in the online table of contents at https://academic.oup.com/cdn/.

Abbreviations used: AGP, α-1 acid glycoprotein; AI, Adequate Intake; CRP, C-reactive protein; DMG, dimethylglycine; HCAZ, head-circumference-for-age *z*-score; LAZ, length-for-age *z*-score; LMIC, low- or middle-income country; MDAT, Malawi Developmental Assessment Tool; MUAC, midupper arm circumference; SNP, single nucleotide polymorphism; TMAO, trimethylamine *N*-oxide; UPLC-MS/MS, ultrahigh performance liquid chromatography–tandem mass spectrometry.

## References

[1] Zeisel SH . The fetal origins of memory: the role of dietary choline in optimal brain development. J Pediatr. 2006;149(5):S131–6. 17212955 10.1016/j.jpeds.2006.06.065 PMC2430654

[2] Headey DD , Alderman HH. The relative caloric prices of healthy and unhealthy foods differ systematically across income levels and continents. J Nutr. 2019;149(11):2020–33. 31332436 10.1093/jn/nxz158 PMC6825829

[3] Wiedeman AM , Barr SI, Green TJ, Xu Z, Innis SM, Kitts DD. Dietary choline intake: current state of knowledge across the life cycle. Nutrients. 2018;10(10):1513. 10.3390/nu10101513 PMC6213596 30332744

[4] Iannotti LL , Lutter CK, Bunn DA, Stewart CP. Eggs: the uncracked potential for improving maternal and young child nutrition among the world' s poor. Nutr Rev. 2014;72(6):355–68. 24807641 10.1111/nure.12107

[5] Iannotti LL , Lutter CK, Stewart CP, Gallegos Riofrío CA, Malo C, Reinhart G, Palacios A, Karp C, Chapnick M, Cox K et al. Eggs in early complementary feeding and child growth: a randomized controlled trial. Pediatrics. 2017;140(1):e20163459. 28588101 10.1542/peds.2016-3459

[6] Iannotti LL , Lutter CK, Waters WF, Riofrío CAG, Malo C, Reinhart G, Palacios A, Karp C, Chapnick M, Cox K et al. Eggs early in complementary feeding increase choline pathway biomarkers and DHA: a randomized controlled trial in Ecuador. Am J Clin Nutr. 2017;106:1382–9. 10.3945/ajcn.117.160515 PMC5698841 29092879

[7] Stewart CP , Caswell B, Iannotti L, Lutter C, Arnold CD, Chipatala R, Prado EL, Maleta K. The effect of eggs on early child growth in rural Malawi: the Mazira Project randomized controlled trial. Am J Clin Nutr. 2019;110(4):1026–33. 31386106 10.1093/ajcn/nqz163 PMC6766435

[8] Prado EL , Maleta KM, Caswell BL, George M, Oakes LM, DeBolt MC, Bragg MG, Arnold CD, Iannotti L, Lutter CK et al. Early child development outcomes of a randomized trial providing one egg per day to children age 6 to 15 months in Malawi. J Nutr. 2020;150(7):1933–42. 32286620 10.1093/jn/nxaa088 PMC7330477

[9] Caswell B , Arnold C, Lutter C, Iannotti L, Chipatala R, Werner E, Maleta K, Stewart C. Impacts of an egg intervention on nutrient intake adequacy among young Malawian children. Matern Child Nutr. 2021;17(3):e13196. 33974324 10.1111/mcn.13196 PMC8189245

[10] Mun JG , Legette LL, Ikonte CJ, Mitmesser SH. Choline and DHA in maternal and infant nutrition: synergistic implications in brain and eye health. Nutrients. 2019;11(5):1125. 10.3390/nu11051125 PMC6566660 31117180

[11] Lutter CK , Caswell BL, Arnold CD, Iannotti LL, Maleta K, Chipatala R, Prado EL, Stewart CP. Impacts of an egg complementary feeding trial on energy intake and dietary diversity in Malawi. Matern Child Nutr. 2021;17(1):e13055. 33128502 10.1111/mcn.13055 PMC7729770

[12] WHO Multicentre Growth Reference Study Group . WHO child growth standards based on length/height, weight and age. Acta Paediatr Suppl. 2006;450:76–85. 16817681 10.1111/j.1651-2227.2006.tb02378.x

[13] Gladstone M , Lancaster GA, Umar E, Nyirenda M, Kayira E, van den Broek NR, Smyth RL. The Malawi Developmental Assessment Tool (MDAT): the creation, validation, and reliability of a tool to assess development in rural African settings. PLoS Med. 2010;7(5):e1000273. 20520849 10.1371/journal.pmed.1000273 PMC2876049

[14] Coates J , Swindale A, Bilinsky P. Household Food Insecurity Access Scale (HFIAS) for measurement of food access: indicator guide. Washington (DC): Food and Nutrition Technical Assistance III Project; 2007.

[15] Wang Z , Levison BS, Hazen JE, Donahue L, Li X-M, Hazen SL. Measurement of trimethylamine-N-oxide by stable isotope dilution liquid chromatography tandem mass spectrometry. Anal Biochem. 2014;455:35–40. 24704102 10.1016/j.ab.2014.03.016 PMC4167037

[16] Erhardt JG , Estes JE, Pfeiffer CM, Biesalski HK, Craft NE. Combined measurement of ferritin, soluble transferrin receptor, retinol binding protein, and C-reactive protein by an inexpensive, sensitive, and simple sandwich enzyme-linked immunosorbent assay technique. J Nutr. 2004;134(11):3127–32. 15514286 10.1093/jn/134.11.3127

[17] Institute of Medicine . Dietary reference intakes for thiamin, riboflavin, niacin, vitamin B6, folate, vitamin B12, pantothenic acid, biotin, and choline. Washington DC: National Academies Press; 1998. 23193625

[18] Fischer LM , da Costa KA, Galanko J, Sha W, Stephenson B, Vick J, Zeisel SH. Choline intake and genetic polymorphisms influence choline metabolite concentrations in human breast milk and plasma. Am J Clin Nutr. 2010;92(2):336–46. 20534746 10.3945/ajcn.2010.29459 PMC2904035

[19] Wiedeman AM , Whitfield KC, March KM, Chen NN, Kroeun H, Sokhoing L, Sophonneary P, Dyer RA, Xu Z, Kitts DD et al. Concentrations of water-soluble forms of choline in human milk from lactating women in Canada and Cambodia. Nutrients. 2018;10(3):381. 10.3390/nu10030381 PMC5872799 29558412

[20] Abratte CM , Wang W, Li R, Axume J, Moriarty DJ, Caudill MA. Choline status is not a reliable indicator of moderate changes in dietary choline consumption in premenopausal women. J Nutr Biochem. 2009;20(1):62–9. 18495456 10.1016/j.jnutbio.2007.12.002

[21] Li Z , Vance DE. Phosphatidylcholine and choline homeostasis. J Lipid Res. 2008;49(6):1187–94. 18204095 10.1194/jlr.R700019-JLR200

[22] Ganz AB , Cohen VV, Swersky CC, Stover J, Vitiello GA, Lovesky J, Chuang JC, Shields K, Fomin VG, Lopez YS et al. Genetic variation in choline-metabolizing enzymes alters choline metabolism in young women consuming choline intakes meeting current recommendations. Int J Mol Sci. 2017;18(2):252. 10.3390/ijms18020252 PMC5343788 28134761

[23] Silver MJ , Corbin KD, Hellenthal G, da Costa K-A, Dominguez-Salas P, Moore SE, Owen J, Prentice AM, Hennig BJ, Zeisel SH. Evidence for negative selection of gene variants that increase dependence on dietary choline in a Gambian cohort. FASEB J. 2015;29(8):3426–35. 25921832 10.1096/fj.15-271056 PMC4511208

[24] Bragg MG , Caswell B, Maleta K, Stewart C. Choline intake in Malawian children aged 6-9 and 12-15 months in an egg intervention trial. Curr Dev Nutr. 2020;4(Suppl 2):816. 10.1093/cdn/nzab150 PMC8881212 35233478

[25] Yakes Jimenez E, Mangani C, Ashorn P, Harris WS, Maleta K, Dewey KG. Breast milk from women living near Lake Malawi is high in docosahexaenoic acid and arachidonic acid. Prostaglandins Leukot Essent Fatty Acids. 2015;95:71–8. 25601798 10.1016/j.plefa.2014.12.002

[26] Martínez-del Campo A , Bodea S, Hamer HA, Marks JA, Haiser HJ, Turnbaugh PJ, Balskus EP. Characterization and detection of a widely distributed gene cluster that predicts anaerobic choline utilization by human gut bacteria. mBio. 2015;6(2):e00042–15. 25873372 10.1128/mBio.00042-15 PMC4453576

[27] Romano KA , Vivas EI, Amador-Noguez D, Rey FE. Intestinal microbiota composition modulates choline bioavailability from diet and accumulation of the proatherogenic metabolite trimethylamine-N-oxide. mBio. 2015;6(2):e02481. 25784704 10.1128/mBio.02481-14 PMC4453578

[28] Yang S , Li X, Yang F, Zhao R, Pan X, Liang J, Tian L, Li X, Liu L, Xing Y et al. Gut microbiota-dependent marker TMAO in promoting cardiovascular disease: inflammation mechanism, clinical prognostic, and potential as a therapeutic target. Front Pharmacol. 2019;10:1360. 31803054 10.3389/fphar.2019.01360 PMC6877687

[29] Haghikia A , Li XS, Liman TG, Bledau N, Schmidt D, Zimmermann F, Kränkel N, Widera C, Sonnenschein K, Haghikia A et al. Gut microbiota-dependent TMAO predicts risk of cardiovascular events in patients with stroke and is related to proinflammatory monocytes. Arterioscler Thromb Vasc Biol. 2018;38(9):2225–35. 29976769 10.1161/ATVBAHA.118.311023 PMC6202215

[30] Andraos S , Jones B, Lange K, Clifford SA, Thorstensen EB, Kerr JA, Wake M, Safferty R, Burgner DP, O'Sullivan JM. Trimethylamine N-oxide (TMAO) is not associated with cardiometabolic phenotypes and inflammatory markers in children and adults. Curr Dev Nutr. 2021;5(1):nzaa179. 33501405 10.1093/cdn/nzaa179 PMC7813154

[31] Wilcox J , Skye SM, Graham B, Zabell A, Li XS, Li L, Shelkay S, Fu X, Neale S, O'Laughlin C et al. Dietary choline supplements, but not eggs, raise fasting TMAO levels in participants with normal renal function: a randomized clinical trial. Am J Med. 2021;134(9):1160–9.e3. 33872583 10.1016/j.amjmed.2021.03.016 PMC8410632

[32] DiMarco DM , Missimer A, Murillo AG, Lemos BS, Malysheva OV, Caudill MA, Blesso CN, Fernandez ML. Intake of up to 3 eggs/day increases HDL cholesterol and plasma choline while plasma trimethylamine-N-oxide is unchanged in a healthy population. Lipids. 2017;52(3):255–63. 28091798 10.1007/s11745-017-4230-9

[33] Zhu C , Sawrey-Kubicek L, Bardagjy AS, Houts H, Tang X, Sacchi R, Randolph JM, Steinberg FM, Zivkovic AM. Whole egg consumption increases plasma choline and betaine without affecting TMAO levels or gut microbiome in overweight postmenopausal women. Nutr Res. 2020;78:36–41. 32464420 10.1016/j.nutres.2020.04.002

[34] West AA , Shih Y, Wang W, Oda K, Jaceldo-Siegl K, Sabaté J, Haddad E, Rajaram S, Caudill MA, Burns-Whitmore B. Egg n-3 fatty acid composition modulates biomarkers of choline metabolism in free-living lacto-ovo-vegetarian women of reproductive age. J Acad Nutr Diet. 2014;114(10):1594–600. 24726349 10.1016/j.jand.2014.02.012

[35] Miller CA , Corbin KD, Da Costa KA, Zhang S, Zhao X, Galanko JA, Blevins T, Bennett BJ, O'Connor A, Zeisel SH. Effect of egg ingestion on trimethylamine-N-oxide production in humans: a randomized, controlled, dose-response study. Am J Clin Nutr. 2014;100(3):778–86. 24944063 10.3945/ajcn.114.087692 PMC4135488

[36] National Statistical Office (NSO) [Malawi], ICF . Malawi demographic and health survey 2015–16. Zomba, Malawi and Rockville (MD): NSO and ICF; 2017.

[37] Münger LH , Garcia-Aloy M, Vázquez-Fresno R, Gille D, Rosana ARR, Passerini A, Soria-Florido MT, Pimentel G, Sajed T, Wishart DS et al. Biomarker of food intake for assessing the consumption of dairy and egg products. Genes Nutr. 2018;13:26. 30279743 10.1186/s12263-018-0615-5 PMC6162878

[38] Thompson FE , Kirkpatrick SI, Subar AF, Reedy J, Schap TE, Wilson MM, Krebs-Smith SM. The National Cancer Institute's dietary assessment primer: a resource for diet research. J Acad Nutr Diet. 2015;115(12):1986–95. 26422452 10.1016/j.jand.2015.08.016 PMC4663113

[39] Noerman S , Kärkkäinen O, Mattsson A, Paananen J, Lehtonen M, Nurmi T, Tuomainen TP, Voutilainen S, Hanhineva K, Virtanen JK. Metabolic profiling of high egg consumption and the associated lower risk of type 2 diabetes in middle-aged Finnish men. Mol Nutr Food Res. 2019;63(5):e1800605. 30548819 10.1002/mnfr.201800605

[40] Stewart CP , Iannotti LL, Lutter CK, Maleta KM. The Mazira Project: an evaluation of eggs during complementary feeding in rural Malawi [Internet]. clinicaltrialsgov identifier. NCT03385252. Available from: https://clinicaltrials.gov/ct2/show/NCT03385252?term=NCT03385252&draw=2&rank=1.

[41] Zeisel SH . Choline nutritional status: development of a biomarker panel [Internet]. clinicaltrialsgov identifier. NCT03726671. Available from: https://clinicaltrials.gov/ct2/results?cond=&term=+NCT03726671&cntry=&state=&city=&dist=.

