# Supplementary material for: Plasma Choline Concentration Was Not Increased After a 6-Month Egg Intervention in 6–9-Month-Old Malawian Children: Results from a Randomized Controlled Trial
Source: Curr Dev Nutr. 2022 Feb 23;6(2):nzab150. doi: 10.1093/cdn/nzab150 (PMC8881212; doi:10.1093/cdn/nzab150)
Supplement: nzab150_Supplemental_File [file nzab150_supplemental_file.docx]

**Plasma choline concentration after a six month egg intervention among 6-9 month old Malawian children: results from a randomized controlled trial**

**Megan G Bragg, Elizabeth L Prado, Charles D Arnold, Kenneth M Maleta, Bess L Caswell, Brian J Bennett, Lora L Iannotti, Chessa K Lutter, Christine P Stewart**

**Supplementary Materials**

Supplemental Figure 1 - Correlation between two measures of plasma choline, betaine, and trimethylamine N-oxide among Mazira Project participants

Supplemental Figure 2 - Histogram of p-values from a semi-quantitative metabolomic linear regression analysis of 689 metabolites by intervention group among Mazira Project participants (n=400)

Supplemental Figure 3 - Volcano plot from a semi-quantitative metabolomic linear regression analysis of 689 metabolites by intervention group among Mazira Project participants (n=400)

Supplemental Table 1 – Baseline characteristics of children enrolled in the Mazira Project and included vs excluded from the current secondary analysis

Supplemental Table 2 – Results from exploratory effect modification analyses of the effect of intervention group assignment on selected choline biomarkers among participants of the Mazira Project (n=400)

Supplemental Table 3 – Exploratory analyses investigating plasma choline and related biomarkers as mediators of the Mazira Project primary results (n=400)

**Supplemental Figure 1 – Correlation between two measures of plasma choline, betaine, and trimethylamine N-oxide among Mazira Project participants^a^**

1. Plasma choline (r = 0.92)


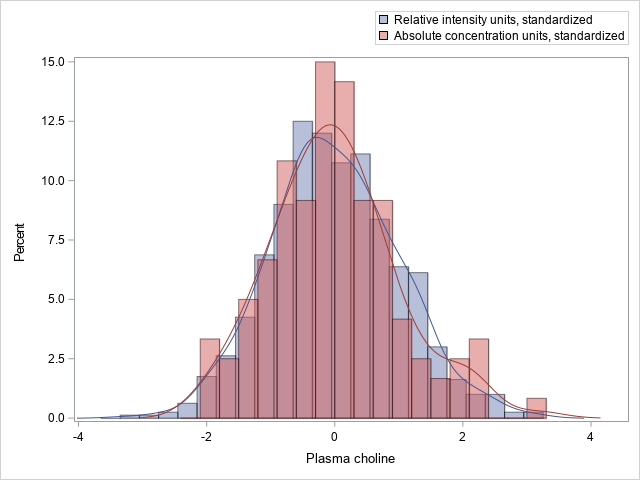

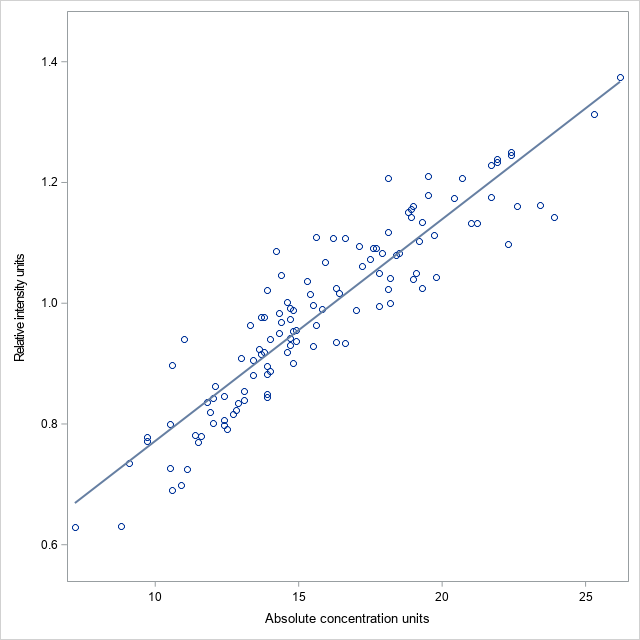


1. Plasma betaine (r = 0.98)


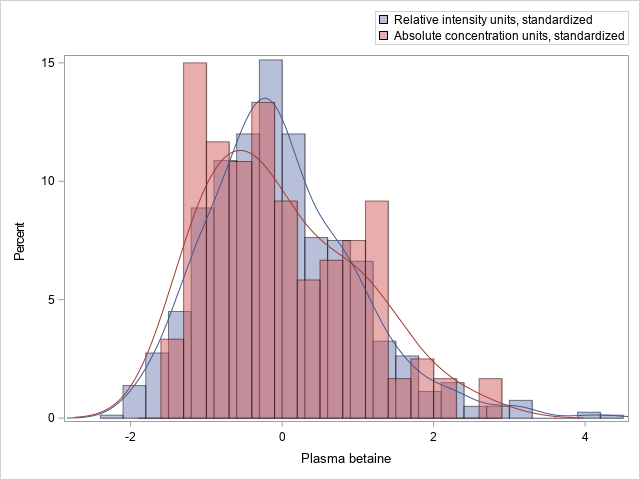

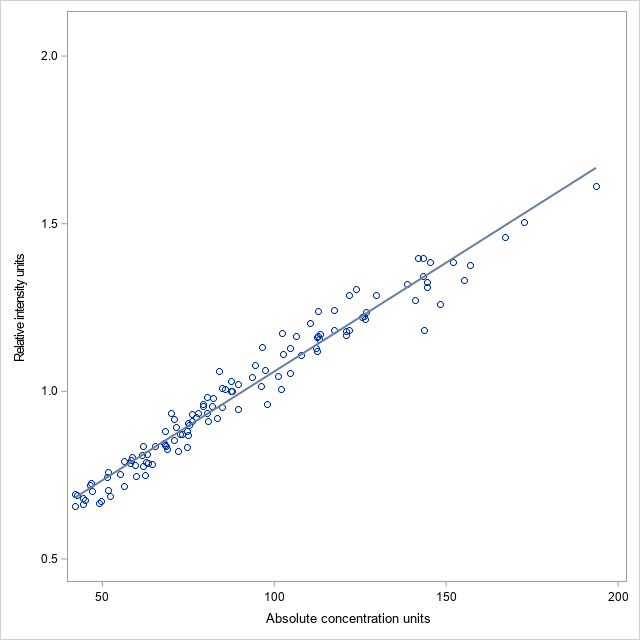


1. Plasma trimethylamine N-oxide (r = 0.98)


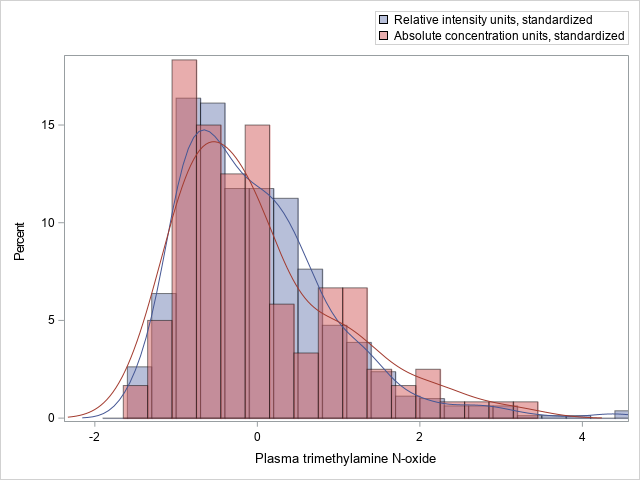

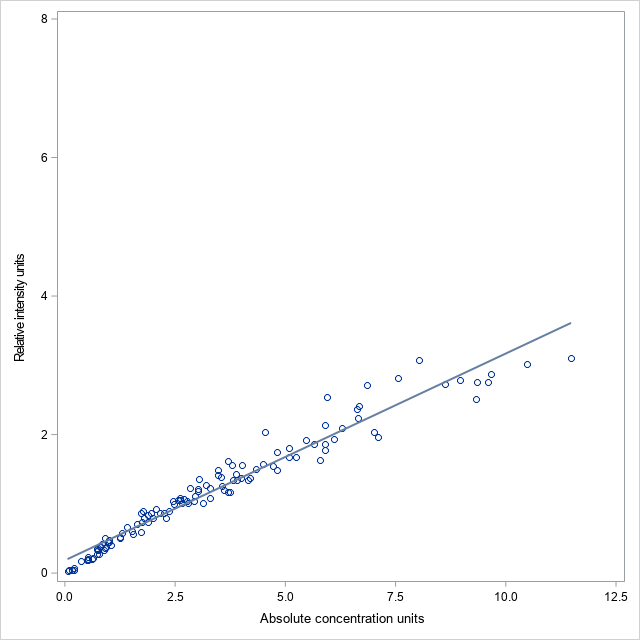


^a^ Relative intensity units were measured using UPLC-MS/MS during metabolomic analysis among 400 participants (200 per group). Absolute concentration units were measured quantitatively using LC-MS/MS among 60 participants (30 per group). Data are standardized for comparison.

**Supplemental Figure 2 – Histogram of p-values from a semi-quantitative metabolomic linear regression analysis of 689 metabolites by intervention group among Mazira Project participants (n=400)^a^**


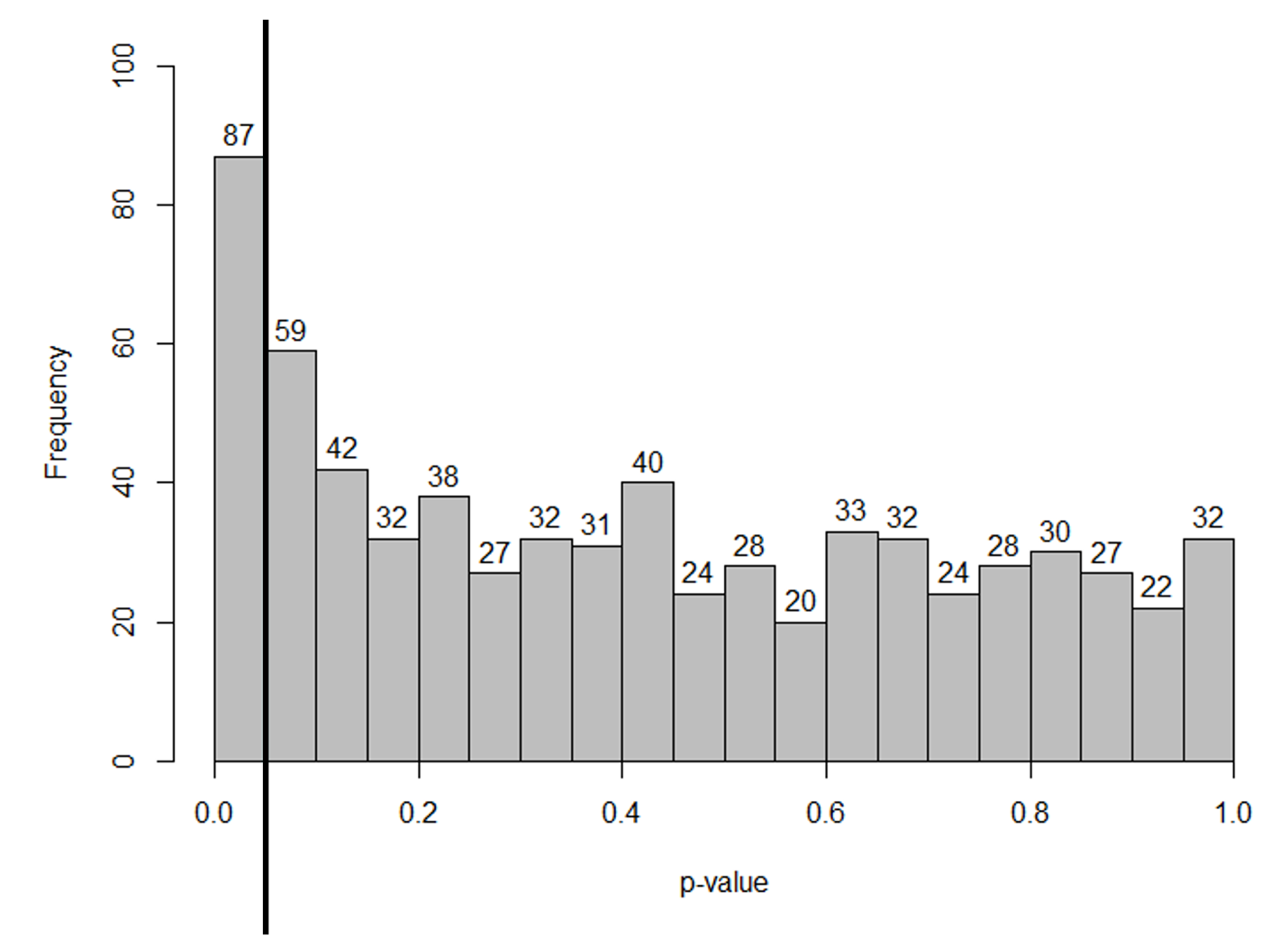


^a^ The black vertical line indicates a p-value of 0.05.

**Supplemental Figure 3 – Volcano plot from a semi-quantitative metabolomic linear regression analysis of 689 metabolites by intervention group among Mazira Project participants (n=400)^a^**


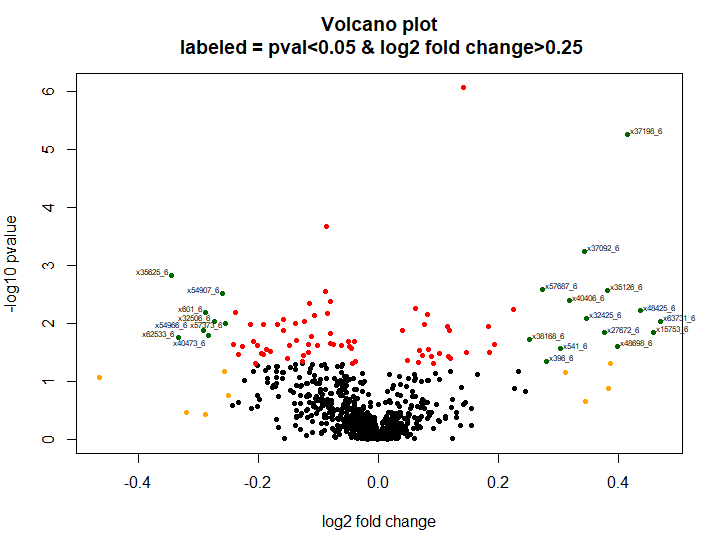


^a^ Each dot represents a single metabolite. Red dots indicate metabolites for which p<0.05, yellow dots indicate metabolites for which log2 fold change >0.25, and green dots indicate metabolites for which p<0.05 and log2 fold change >0.25. Green metabolites are labeled with their assigned codes. Individual metabolite names are provided in Table 4.

**Supplemental Table 1 – Baseline characteristics of children enrolled in the Mazira Project and included vs excluded from the current secondary analysis**

|  | Included (n= 400) | Excluded (n= 260)^a^ | p value |
| --- | --- | --- | --- |
|  | Mean (SD) or (%) | Mean (SD) or (%) |  |
| *Child and maternal characteristics* |  |  |  |
| Child age (mo) | 7.4 (1.2) | 7.4 (1.2) | 0.849 |
| Male | 53.3 | 49.2 | 0.313 |
| First born | 27.8 | 27.3 | 0.886 |
| Animal source food consumption^b^ |  |  |  |
| Consumed dairy | 9.8 | 6.5 | 0.145 |
| Consumed meat | 2.5 | 0.8 | 0.103 |
| Consumed egg | 3.8 | 4.6 | 0.588 |
| Consumed fish | 23.6 | 32.7 | **0.010** |
| Any breastmilk | 99.7 | 100.0 | 0.419 |
| Anemia prevalence (Hgb < 11 g/dL) | 62.2 | 57.8 | 0.320 |
| Malaria prevalence | 14.5 | 8.6 | **0.042** |
| Prevalence of stunting (LAZ ≤ -2) (%) | 13.8 | 13.5 | 0.916 |
| Prevalence of wasting (WLZ ≤ -2) (%) | 1.5 | 0.4 | 0.172 |
| Prevalence of underweight (WAZ ≤ -2) (%) | 8.3 | 7.3 | 0.661 |
| Prevalence of small head size (HCAZ ≤ -2) (%) | 23.5 | 15.1 | **0.008** |
| Prevalence of developmental delay (%)^c^ |  |  |  |
| Gross motor | 0.3 | 0.0 | 0.416 |
| Fine motor | 2.5 | 2.7 | 0.914 |
| Personal social | 1.5 | 2.3 | 0.471 |
| Language | 0.5 | 0.8 | 0.679 |
| Maternal age (y) | 26.0 (6.8) | 25.9 (6.7) | 0.857 |
| Maternal BMI (kg/m2) | 21.8 (3.0) | 21.9 (3.0) | 0.445 |
| Mother completed primary school (%) | 20.5 | 19.1 | 0.656 |
| Mother can read (%) | 47.7 | 42.9 | 0.228 |
|  |  |  |  |
| *Household characteristics* |  |  |  |
| Number of household members | 6.0 (2.6) | 5.7 (2.7) | 0.242 |
| Moderate to severe food insecurity^d^ | 78.0 | 77.7 | 0.926 |
| Owns latrine | 96.5 | 96.3 | 0.918 |
| Owns cows | 2.5 | 3.7 | 0.399 |
| Owns goats | 20.0 | 17.3 | 0.388 |
| Owns chickens | 33.3 | 31.0 | 0.552 |
| Less than 10 minutes to water source (%) | 53.5 | 59.2 | 0.160 |

^HCZ – head circumference-for-age z-score; Hgb – hemoglobin; LAZ – length-for-age z-score; WAZ – weight-for-age z-score; WLZ – weight-for-length z-score^

^a^ Children enrolled in the main trial were excluded from this analysis if they were missing one or both blood samples (n=147) or were not randomly selected for biochemical analysis (n=113)

^b^ As reported by the caregiver on a 24-hour dietary recall

^c^ As defined by the Malawi Developmental Assessment Tool (MDAT)^11^

^d^ As defined by the Household Food Insecurity Access Scale^26^

**Supplemental Table 2 – Results from exploratory effect modification analyses^a^ of the effect of group assignment on choline biomarkers among Mazira Project participants (n=400)**

|  | p for interaction |
| --- | --- |
| *Plasma choline* |  |
| Child sex (male vs female) | 0.410 |
| First born (first vs not first) | 0.163 |
| Household food insecurity (mild/none vs moderate/severe) | 0.619 |
| Housing and asset score (quintile 1 vs quintiles 2-5) | 0.703 |
| Baseline length-for-age z-score (below vs above <-1) | 0.576 |
| Maternal age (below vs above 20y) | 0.180 |
| Maternal education (incomplete primary vs primary or greater) | 0.870 |
| *Plasma betaine* |  |
| Child sex | 0.908 |
| First born | 0.582 |
| Household food insecurity | 0.215 |
| Housing and asset score | 0.821 |
| Baseline length-for-age z-score | 0.773 |
| Maternal age | 0.404 |
| Maternal education | 0.607 |
| *Log plasma dimethylglycine* |  |
| Child sex | 0.381 |
| First born | 0.759 |
| Household food insecurity | 0.835 |
| Housing and asset score | 0.180 |
| Baseline length-for-age z-score | 0.815 |
| Maternal age | 0.784 |
| Maternal education | 0.637 |
| *Log plasma trimethylamine N-oxide* |  |
| Child sex | 0.100 |
| First born | 0.981 |
| Household food insecurity | 0.514 |
| Housing and asset score | 0.887 |
| Baseline length-for-age z-score | 0.251 |
| Maternal age | 0.858 |
| Maternal education | 0.687 |
| *Plasma docosahexaenoic acid* |  |
| Child sex | 0.500 |
| First born | 0.438 |
| Household food insecurity | 0.531 |
| Housing and asset score | 0.747 |
| Baseline length-for-age z-score | 0.209 |
| Maternal age | 0.550 |
| Maternal education | 0.864 |

^a^ Each moderator was made dichotomous and tested in regression models adjusted for baseline biomarker value.

**Supplemental Table 3 – Exploratory analyses^a^ investigating plasma choline and related biomarkers as mediators of the Mazira Project primary results (n=400)**

|  | Total Effect^b^ | Direct Effect | Indirect Effect |
| --- | --- | --- | --- |
| Fine motor delay |  |  |  |
| Choline | -4.6 (-11.4, 2.2) | -4.6 (-11.2, 2.3) | 0.03 (-0.3, 0.3) |
| Betaine |  | -4.6 (-11.4, 2.2) | 0.05 (-0.6, 0.7) |
| Log DMG |  | -4.6 (-11.4, 2.3) | -0.01 (-0.1, 0.1) |
| Log TMAO |  | -5.1 (-11.9, 1.7) | 0.5 (-0.4, 1.5) |
| DHA |  | -4.5 (-11.3, 2.3) | -0.09 (-0.6, 0.4) |
|  |  |  |  |
| Head circumference-for-age z-score |  |  |  |
| Choline | 24.6 (2.1, 47.2) | 27.4 (5.1, 49.6) | -2.7 (-7.0, 1.6) |
| Betaine |  | 24.5 (2.0, 4.7) | 0.1 (-0.6, 0.9) |
| Log DMG |  | 24.9 (2.5, 47.2) | -0.2 (-3.0, 2.6) |
| Log TMAO |  | 24.6 (1.9, 47.4) | 0.04 (-2.9, 2.9) |
| DHA |  | 24.7 (2.1, 47.3) | -0.04 (-0.8, 0.7) |

^DHA – docosahexaenoic acid; DMG – dimethylglycine; TMAO – trimethylamine N-oxide^

^a^ Mediation analyses were performed using structural equation modeling to test for mediation of the two primary findings of the Mazira Project (fine motor delay, head circumference-for-age z-score) by plasma choline and related biomarkers.

^b^ Total effects are presented as the difference in means (95% CI) between intervention and control in outcome variables. Direct effects are the effect of intervention group after controlling for the mediator. Indirect effects are the effect of intervention group that is acting through choline/mediator (in SD units for choline, betaine, DHA; in log units for DMG and TMAO).
